# Clinicopathological characteristics of co-existing or mixed colorectal cancer and neuroendocrine tumor: Report of five cases

**DOI:** 10.1515/biol-2022-0774

**Published:** 2023-12-12

**Authors:** Ling Zhang, Xiaoling Wang, Yun Wang, Yan Zeng, Li Li

**Affiliations:** Department of Pathology, Guang’anmen Hospital, China Academy of Chinese Medical Sciences, Beijing 100053, China; Department of Radiology, Guang’anmen Hospital, China Academy of Chinese Medical Sciences, Beijing 100053, China

**Keywords:** colorectum, adenocarcinoma, neuroendocrine tumor, co-existing or mixed type

## Abstract

Coexisting or mixed type of colorectal tumors has been rarely reported. This study was designed to investigate clinicopathological characteristics of co-existing or mixed colorectal adenocarcinoma and highly differentiated neuroendocrine tumor (NET-G1). To do that, clinicopathological characteristics of five cases of co-existing or mixed colorectal adenocarcinoma and NET-G1 admitted to our institution between 2017 and 2021 were retrospectively analyzed and literature review was conducted. Four patients were male and one female, aged 62–75 years old. Among them, four cases were diagnosed with rectal cancer and one case of colon cancer. Gross examination found that one patient was diagnosed with multiple colon polyps including three malignant polyps, and the remaining four cases of ulcerous masses. The tumors infiltrated into the muscle layer in two cases, and three cases with tumors infiltrating into surrounding adipose tissues. Microscopic examination revealed one patient developed poorly differentiated adenocarcinoma and four cases of moderately differentiated adenocarcinoma. Four patients had adenocarcinoma and NET-G1 in colon, and one case of adenocarcinoma in colon and NET-G1 in appendix. To conclude, co-existing or mixed colorectal tumors are extremely rare in clinical settings. Clinicopathological characteristics of five cases of co-existing or mixed adenocarcinoma and NET-G1 are diverse and adenocarcinoma is more aggressive in most affected patients.

## 1 Introduction

Coexisting or mixed type of colorectal tumors has been rarely reported in clinical practice. The co-existing or mixed type masses can be composed of adenocarcinoma and non-neuroendocrine tumors, such as ovarian granulosa cell tumor [1], malignant lymphoma [2], malignant melanoma [3], carcinosarcoma [4], and even leukemia [5]. In particular, the incidence of coexisting or mixed type of colorectal adenocarcinoma and neuroendocrine tumors (NET) is extremely low [6].

In the present clinical trial, we reported the clinicopathological characteristics of five cases of coexisting or mixed adenocarcinoma and NET-G1. In one patient, NET-G1 accounted for 40% of the whole tumor, and less than 10% in the remaining four cases, respectively. Two patients presented with the collision of the colorectal adenocarcinoma and the NET-G1, and the colorectal adenocarcinoma was adjacent to the NET-G1 in the other three patients, aiming to supplement clinical evidence for in-depth understanding of this rare comorbid condition.

## 2 Case presentation

### 2.1 Baseline data

Clinicopathological data of five patients who were pathologically diagnosed with co-existing or mixed colorectal adenocarcinoma and NET-G1 in the Department of Pathology of Guang’anmen Hospital of China Academy of Chinese Medical Sciences from 2017 to 2021 were retrospectively analyzed, as shown in Table 1. The specimens were fixed with 4% neutral formaldehyde, embedded in paraffin, and the section was 4 μm in thickness and then subject to HE staining. Immunohistochemical staining was performed by EnVision method. Antibodies CD56, Syn, CgA, Ki-67, and kits were purchased from ZSGB-BIO (Beijing, China). Positive and negative controls were set for each antibody. Immunohistochemical staining was performed using a fully automatic Leica BOND MAX staining robot (Leica Microsystems, Wetzlar, Germany). Clinical baseline data are illustrated in Table 1.

**Table 1:** Clinical baseline data of five patients

Sex	Age	Chief complaint	Enteroscopic examination	Preoperative diagnosis	Preoperative pathological biopsy	Treatment regimen	Postoperative survival
Female	69	Intermittent hematochezia	Single ulcer mass 1.6 cm in diameter	Rectal cancer	Moderately differerntiated adenocarcinoma	Preoperative neoadjuvant chemotherapy (*n* = 3) + radical surgery + postoperative chemotherapy (*n* = 3)	4 months
Male	63	Left lower abdomen pain with recurrent hematochezia for 6 months and worsened for 4 days	Single ulcer mass 4 cm in diameter	Rectal cancer	Poorly differerntiated adenocarcinoma	Radical surgery + postoperative chemotherapy (*n* = 6)	14 months
Male	72	Hematochezia and thinning stool for over 1 month	Single ulcer mass 4 cm in diameter	Rectal cancer	Moderately differerntiated adenocarcinoma	Preoperative neoadjuvant chemotherapy (*n* = 1) + radical surgery + postoperative chemotherapy (*n* = 6)	32 months
Male	63	Intermittent hematochezia and irregular stool for 2 months	Three polypoid masses, 4.5, 4, and 2 cm in diameter	Colon cancer	Moderately differerntiated adenocarcinoma	Radical surgery + postoperative chemotherapy (*n* = 4)	33 months
Male	75	Increased stool frequency complicated with mild hematochezia for 6 months	Single ulcer mass 6 cm in diameter	Rectal cancer	Moderately differerntiated adenocarcinoma	Radical surgery + postoperative chemotherapy (*n* = 5)	53 months


**Informed consent:** Informed consent has been obtained from all individuals included in this study.
**Ethical approval:** The research related to human use has been complied with all the relevant national regulations, institutional policies and in accordance with the tenets of the Helsinki Declaration, and has been approved by the authors’ institutional review board or equivalent committee.

### 2.2 HE staining

**Figure 1:**
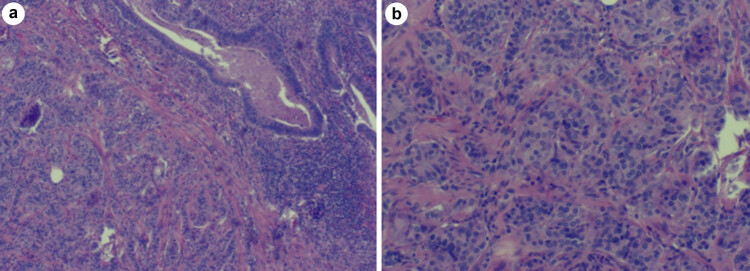
Adenocarcinoma is seen in the upper right corner and NET-G1 in the lower left corner, which are independent with no migration (a, 4 × 10); NET-G1 is observed in small nest, strip, round, or oval shape with uniform size (b, 10 × 10).

Of the five patients, NET-G1 was located at the lower incisional margin in two cases, located in the appendix in one case, adjacent to the adenocarcinoma in one case, and located within the adenocarcinoma in one case, respectively. The tumors of five patients were all composed of two components. One component consisted of adenocarcinoma which was arranged in glandular tubular and sieve shape, and the tumor cells were in columnar or short cubic shape, with large and abnormal nuclei and mitotic count. The other component was observed in small nest, beam, or strip shape, with round or oval nucleus in uniform size, with slight abnormity, which was pathologically diagnosed as NET-G1. These two structures were mutually independent. In one case, NET-G1 volume accounted for 40% of the whole tumor and less than 10% in the remaining four cases. No excessive migration of cells or structures was found, as demonstrated in Figure 1.

### 2.3 Immunohistochemical staining

**Figure 2:**
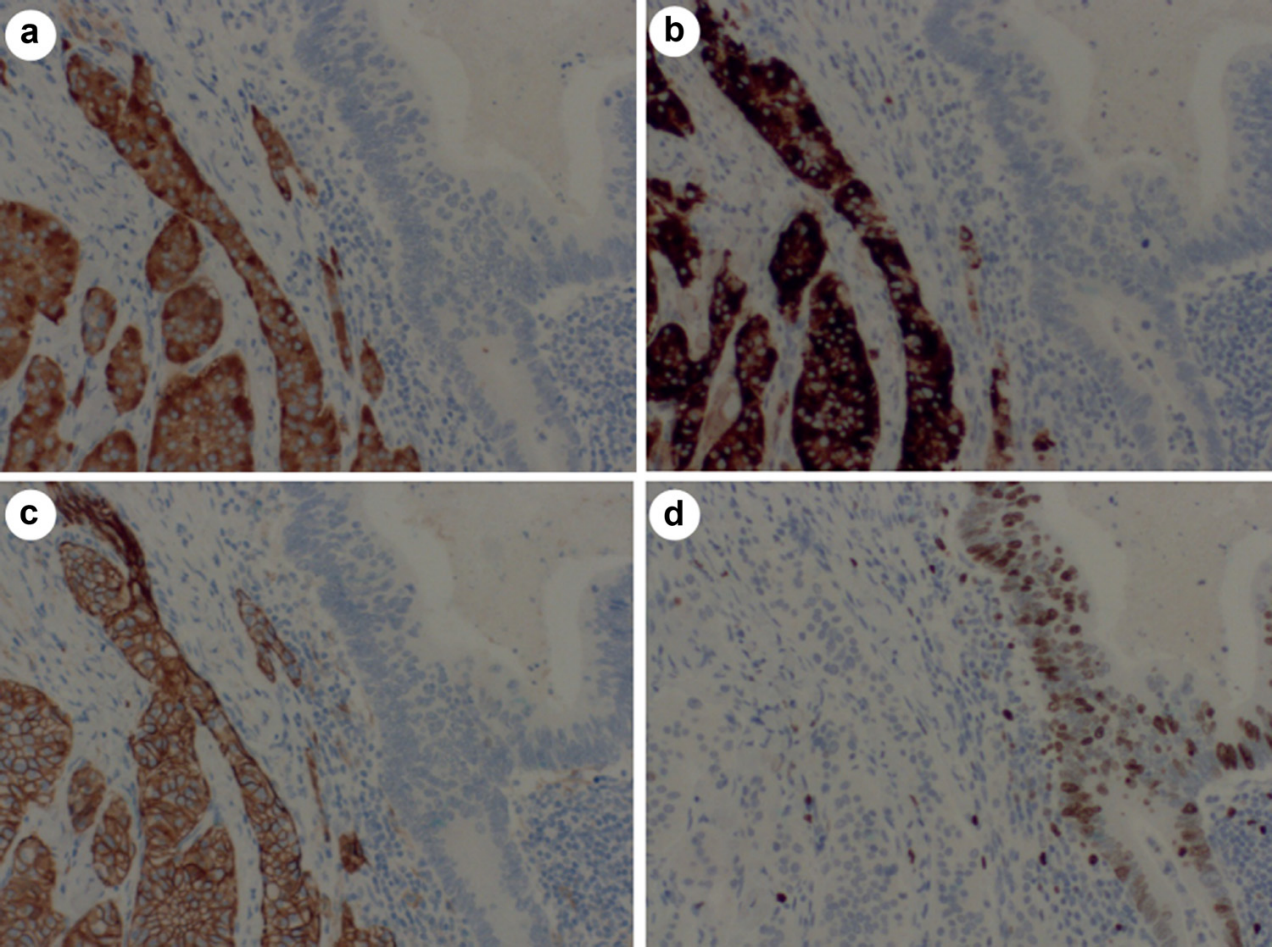
Adenocarcinoma in upper right corner tested negative for Syn, and NET-G1 positive for Syn in the lower left corner (a); adenocarcinoma in upper right corner tested negative for CgA and NET-G1 positive for CgA in the lower left corner (b); adenocarcinoma in upper right corner tested negative for CD56 and NET-G1 positive for CD56 in the lower left corner (c); and adenocarcinoma in upper right corner tested positive for Ki-67 of 40–50% and NET-G1 positive for Ki-67 of <1% in the lower left corner (d, 10 × 10).

Among five cases, NET-G1 was tested positive for Syn, CgA, and CD56 (Figure 2a–c). NET-G1 was tested positive for Ki-67 of <1% (Figure 2d). In addition, adenocarcinoma was tested negative for Syn, CgA, and CD56 (Figure 2a–c), respectively, and tested positive for Ki-67 of 40–50%. (Figure 2d).

### 2.4 Postoperative follow-up

The overall survival was ranged from 4, 14, 32, and 33 to 53 months, respectively. During postoperative follow-up, one patient who developed lung metastasis before radical resection of rectal cancer underwent surgical resection of lung tumor after radical surgery. Postoperative pathological examination confirmed the diagnosis of adenocarcinoma, whereas no postoperative metastasis was detected in the remaining four cases.

## 3 Discussion

Co-existing primary tumors are rarely encountered, and co-existing or mixed NET and non-NET are even rarer in clinical practice. According to the release data of the European Network for Rare Cancer Registry in 2008, the annual incidence of mixed NET is 1 out of 10 million cases, and only 96 cases can survive. Moreover, mixed NET is mostly reported as individual cases and small-scale retrospective studies. Due to the extremely rare incidence, the quality of published data is poor, and no consensus has been reached regarding the inconsistent terminology, epidemiology, prognosis, and optimal treatment regimen [6].

Mixed NET and non-NET are defined as tumors at least comprising two types of tumor cells with different morphologies, including one type of neuroendocrine and the other type of non-neuroendocrine components, mainly adenocarcinoma, occasionally squamous cell carcinoma or sarcoma, etc. [7,8] Mixed epithelial tumors and neuroendocrine components are rare, accounting for approximately 1–2% of all malignant colorectal tumors [9,10]. Cordier [11] reported the first case of mixed gastrointestinal NET and exocrine gland tumor in 1924. Nevertheless, descriptive languages and terminologies led to widespread confusion. Lewin demonstrated that carcinoid tumors contain mixed glandular-endocrine cell carcinomas and identified mixed glandular-endocrine cell carcinomas from the histopathological perspective. Therefore, he became the first to propose the definition of mixed or composite adenoneuroendocrine tumors and subdivide them into composite tumor, collision tumor, and amphicrine tumor [12]. In 2000, NET was classified into mixed exocrine and endocrine carcinomas according to the WHO classification criteria and defined that each tumor component accounted for at least 30% [13]. Mixed adenocarcinoma and NET are extremely rare in clinical practice, and the proportion of each tumor component significantly differs ranging from 1 to 99%, which can occur in almost all organs [14].

The mechanism underlying co-existing or mixed colorectal cancer remains elusive. At present, three main hypotheses have been proposed regarding its origin. First, epithelial and endocrine components originate from corresponding precursor cells in a synchronous or asynchronous manner. Second, two types of tumors are derived from the same category of pluripotent stem cells, which can differentiated in a bidirectional manner during the process of canceration. Third, two categories of tumors originate from the same pluripotent stem cells and gradually transform from non-neuroendocrine epithelial cells into neuroendocrine cells due to gene mutation or micro-environmental changes [15]. The second and third hypotheses are favored by most researchers and clinicians, supporting that gastrointestinal pluripotent stem cells are transformed into endocrine cells rather than neural crest cells as previously thought [16].

Co-existing or mixed adenocarcinoma and NET-G1 primarily comprise two structures. One component consists of adenocarcinoma arranged in glandular tubular and sieve shape, and the tumor cells are in columnar or short cubic shape, with large and abnormal nuclei and mitotic count. The other NET-G1 component is observed in small nest, beam or strip shape, with round or oval nucleus in uniform size, with slight abnormity. These two structures are mutually independent without migration.

NET is a rare tumor, accounting for 1% of digestive tract malignant tumors [17]. In 2017, NET was classified into NET (G1, G2, G3), NET (G3), and mixed NET by WHO according to the proliferation index and mitotic count [7]. Generally, NET can be manifested in nested, beam, cord, or chrysanthemum shape. In the present study, NET in five cases was observed in small-nest, cord, round, and oval shape, with the same size, mitotic count of <2/10HPF and Ki-67 increment index of <1%. Hence, the diagnosis of NET-G1 was confirmed. Among five patients, one case developed poorly differentiated adenocarcinoma and moderately differentiated adenocarcinoma in the other four cases. The cancer cells were seen in columnar or short cubic shape, with evident dysplasia, accompanied by mitotic count and mucus exudation. Adenocarcinoma and NET-G1 significantly differ in morphology. Adenocarcinoma cells and structures are significantly abnormal, and necrosis and mitosis are commonly noted, whereas NET-G1 cells exhibit mild morphological features. Syn, CgA, and CD56 are diagnostic markers for NET by using immunohistochemical staining.

In this study, all five patients were diagnosed with adenocarcinoma by colonoscopy biopsy. After radical surgical resection, the general morphology resembled that of intestinal cancer, including four cases of ulcer-like and one case of polypoid type. Pathological examination mainly confirmed the diagnosis of adenocarcinoma. However, an experienced pathologist found NET-G1 under the microscopy, which accounted for 40% of the whole tumor in one case and less than 10% in the other four cases. Among them, one patient had lung adenocarcinoma metastasis, and no NET metastasis was detected in any patient, which is consistent with the findings of de Mestier and Cros [10] that moderate-grade mixed NET consists of non-NET and well-differentiated NET, and clinical prognosis mainly depends on the non-NET with high malignancy. According to the WHO, mixed tumor is defined as the proportion of each tumor component should exceed 30%, because the proportion of less than 30% cannot affect the biological behavior of the entire tumor. Nevertheless, it is an arbitrary threshold, which has not been supported by clinical relevance and pathogenic significance [18]. Li et al. reported 23 cases of mixed colorectal adenocarcinoma and NET. Among 23 cases, NET accounted for 15% of the whole tumor in 1 case, and both adenocarcinoma and NET metastasized to the liver. The proportion of NET was <2% in one case. The patient died from NET metastasis alone [19]. Therefore, whether the proportion of single component of mixed tumors is less than 30% should be highlighted remains to be further elucidated. In the present study, the degree of malignancy of adenocarcinoma is higher compared with that of NET-G1. Consequently, the clinical prognosis of the patients primarily depends upon adenocarcinoma. Clinicopathological features of these five cases can provide more evidence for this rare disease. Nevertheless, due to the small sample size and single-center study design, these conclusions remain to be validated by subsequent clinical trials.
